# On Extrapolating Past the Range of Observed Data When Making Statistical Predictions in Ecology

**DOI:** 10.1371/journal.pone.0141416

**Published:** 2015-10-23

**Authors:** Paul B. Conn, Devin S. Johnson, Peter L. Boveng

**Affiliations:** National Marine Mammal Laboratory, NOAA, National Marine Fisheries Service, Alaska Fisheries Science Center, 7600 Sand Point Way NE, Seattle, WA 98115 United States of America; CEFE, FRANCE

## Abstract

Ecologists are increasingly using statistical models to predict animal abundance and occurrence in unsampled locations. The reliability of such predictions depends on a number of factors, including sample size, how far prediction locations are from the observed data, and similarity of predictive covariates in locations where data are gathered to locations where predictions are desired. In this paper, we propose extending Cook’s notion of an independent variable hull (IVH), developed originally for application with linear regression models, to generalized regression models as a way to help assess the potential reliability of predictions in unsampled areas. Predictions occurring inside the generalized independent variable hull (gIVH) can be regarded as interpolations, while predictions occurring outside the gIVH can be regarded as extrapolations worthy of additional investigation or skepticism. We conduct a simulation study to demonstrate the usefulness of this metric for limiting the scope of spatial inference when conducting model-based abundance estimation from survey counts. In this case, limiting inference to the gIVH substantially reduces bias, especially when survey designs are spatially imbalanced. We also demonstrate the utility of the gIVH in diagnosing problematic extrapolations when estimating the relative abundance of ribbon seals in the Bering Sea as a function of predictive covariates. We suggest that ecologists routinely use diagnostics such as the gIVH to help gauge the reliability of predictions from statistical models (such as generalized linear, generalized additive, and spatio-temporal regression models).

## Introduction

In ecology and conservation, a common goal is to make predictions about an unsampled random variable given a limited sample from the target population. For instance, given a model (M), estimated parameters ($\hat{\theta }$), and a covariate vector **x**_*i*_, we often desire to predict a new observation *y*_*i*_ at *i* (where *i* can be a design point or a spatial location). For instance, we might use a generalized linear model (GLM; [1]) or one of its extensions to predict species density or occurrence in a new location. Such predictions can be of direct use to conservation and management, for instance, in estimating population abundance or distribution, and for projecting shifts in species range as a function of climate change. Spatially explicit estimates of abundance are also useful for testing theory related to biogeography or biodiversity (e.g., neutral theory; [2]), and for accurately estimating the strength of density dependence [3].

Early in their training, ecologists and statisticians are warned against extrapolating statistical relationships past the range of observed data. This caution is easily interpreted in the context of single-variable linear regression analysis; one should be cautious in using the fitted relationship to make predictions at some new response *y*_*i*_ whenever *x_i_* < min(**x**) or *x_i_* < max(**x**) (where *x*_*i*_ is an independent variable measured at point *i*). But what about more complicated situations where there are multiple explanatory variables, or when one uses a spatial regression model to account for the residual spatial autocorrelation that is inevitably present in patchy ecological data [4]? How reliable are spatially- or temporally-explicit predictions in sophisticated models for animal abundance and occurrence?

Statisticians have long struggled with the conditions under which fitted regression models are capable of making robust predictions at new combinations of explanatory variables. The issue is sometimes considered more of a philosophical problem than a statistical one, and has even been likened to soothsaying [5]. In our view, the reliability of predictions from statistical models is likely a function of several factors, including (i) the intensity of sampling, (ii) spatial or temporal proximity of the prediction location to locations where there are data, (iii) variability of the ecological process, and (iv) the similarity of explanatory covariates in prediction locations when compared to the ensemble of covariates for observed data locations.

In this paper, we investigate one possibility for defining extrapolation in the GLM and its extensions, including generalized additive models (GAMs; [6, 7]) and spatio-temporal regression models (STRMs). In particular, we exploit some of the same ideas used in multiple linear regression regarding leverage and outliers [8] to operationally define “extrapolation” as making predictions that occur outside of a generalized independent variable hull (gIVH) of observed data points. Application of the gIVH and related criterion (e.g., prediction variance) can provide intuition regarding the reliability of predictions in unobserved locations, and can aid in model construction and survey design. We illustrate use of the gIVH on simulated count data, and on several species distribution model (SDM) formulations for ribbon seals (*Histriophoca fasciata*) in the eastern Bering Sea. In particular, we examine the performance of the gIVH in identifying problematic extrapolations when modeling survey counts using GAMs, GLMs, and STRMs.

## Materials and Methods

All data collected and research activities described in this manuscript were performed under National Marine Fisheries Service research permit number 15126.

### Generalizing the independent variable hull

Extrapolation is often distinguished from interpolation. In a prediction context, we might define (admittedly quite imprecisely) that extrapolation consists of making predictions that are “outside the range of observed data” while interpolation consists of making predictions “inside the range of observed data.” But what exactly do we mean by “outside the range of observed data”? Predictions outside the range of observed covariates? Predictions for locations that are so far (in either geographical or covariate space) from places where data are gathered that we are skeptical that the estimated statistical relationship still holds? To help guide our choice of an operational definition, we turn to early work on outlier detection in simple linear regression analysis.

In the context of outlier detection, Cook [8] defined an independent variable hull (IVH) as the smallest convex set containing all design points of a full-rank linear regression model. Linear regression models are often written in matrix form; that is,

Y=Xβ+ϵ,

where **Y** are observed responses, **X** is a so-called design matrix that includes explanatory variables [9], and ***ϵ*** represent normally distributed residuals (here and throughout the paper, bold symbols will be used to denote vectors and matrices). Under this formulation, the IVH is defined relative to the hat matrix, **V**_LR_ = **X**(**X**′**X**)^−1^ **X**′ (where the subscript “LR” denotes linear regression). Letting *v* denote the maximum diagonal element of **V**_*LR*_ (i.e., *v* = max(diag(**V**_*LR*_))), one can examine whether a new design point, **x**_0_ is within the IVH. In particular, **x**_0_ is within the IVH whenever

$$x_{0}^{\prime }{\left(X^{\prime }X\right)}^{-1}x_{0}\leq v.$$ (1)

Cook [8] used this concept to identify influential observations and possible outliers, arguing that design points near the edge of the IVH are deserving of special attention. Similarly, points outside the IVH should be interpreted with caution.

**Fig 1:**
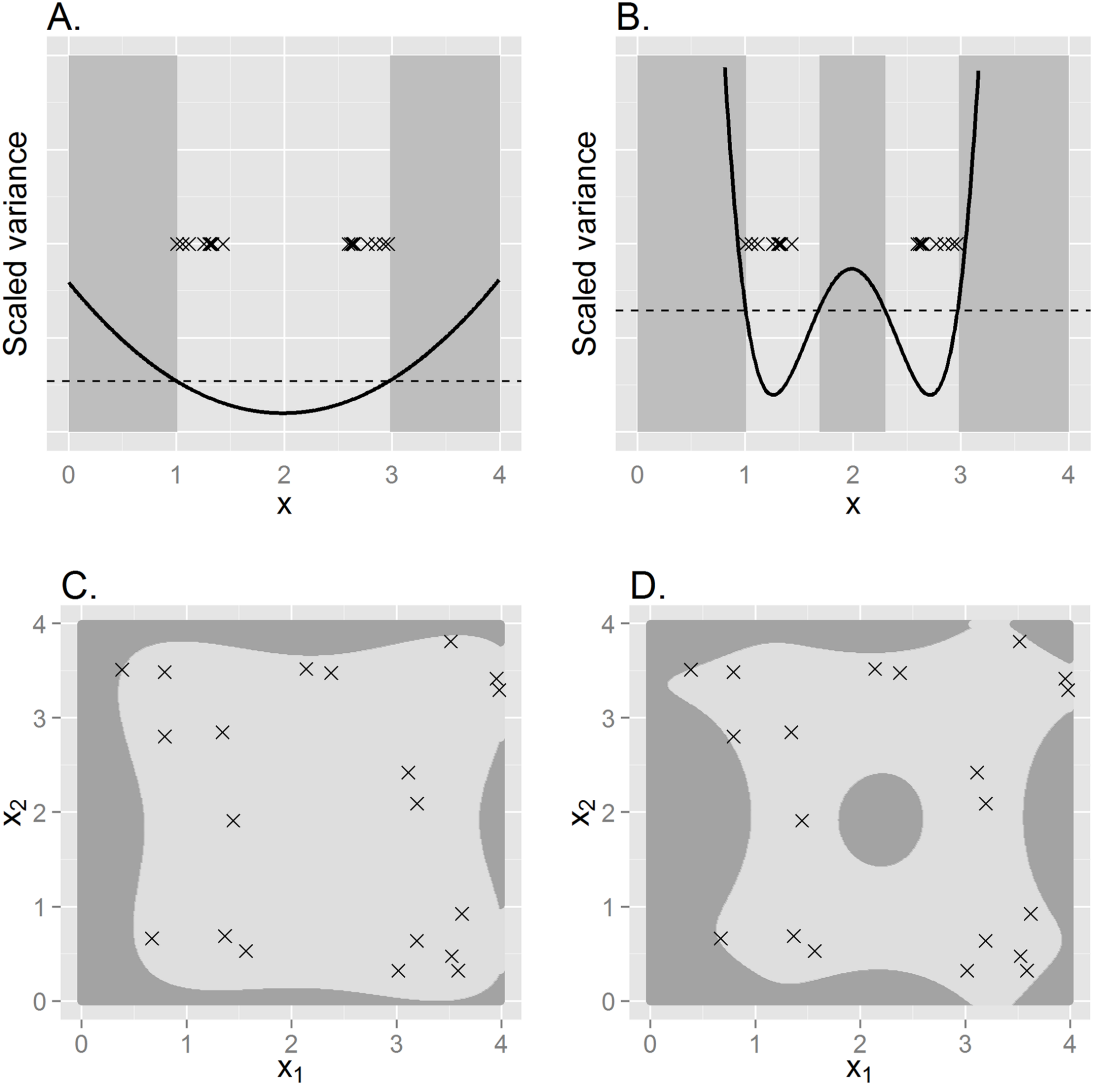
Example IVHs constructed from simulated data. In (A) and (B), linear regression is used to relate a response variable to a single covariate, *x*, obtained at locations denoted with an “x”. Using *x* as a simple linear effect (A), only predictions less than the minimum observed value of *x* or greater than the maximum value of *x* are outside the IVH (shaded area), as scaled prediction variance in these areas (solid line) is greater than the maximum scaled prediction variance for observed data (dashed line). Using both linear and quadratic effects (B), some intermediate points are also outside the IVH. When both linear and quadratic effects of two covariates (*x*_1_ and *x*_2_) are modeled, the IVH is more nuanced and depends on whether interactions are omitted (C) or included (D).

We simulated two sets of design data to help illustrate application of the IVH (Fig 1). In simple linear regression with one predictor variable, predictions on a hypothetical response variable obtained at covariate values slightly outside the range of observed data are also outside the IVH. However, fitting a quadratic model exhibits more nuance; if there is a large gap between design points, intermediate covariate values may also be outside of the IVH and thus more likely to result in problematic predictions. Fitting a model with two covariates and both linear and quadratic effects, the shape of the IVH is somewhat more irregular, and even includes a hole in the middle of the surface when interactions are modeled (Fig 1). These simple examples highlight the sometimes counterintuitive nature of predictive inference, a problem that can only become worse as models with more dimensions are contemplated (including those with temporal or spatial structure). Fortunately, the ideas behind the IVH provide a potential way forward.

Cook’s [8] formulation for the IVH is particular to linear regression analysis, which assumes independent and identically distributed (*iid*) Gaussian error. Thus, it is not directly applicable to generalized models, such as those including alternative response distributions (e.g., Poisson, binomial) or spatial random effects. Further, the hat matrix is not necessarily well defined for more complicated models with prior distributions on parameters, as with hierarchical models. However, since the hat matrix is proportional to prediction variance, Cook [8] notes that design points with maximum prediction variance will be located on the boundary of the IVH. We therefore define a generalized independent variable hull (gIVH) as the set of all predicted locations $S_{0}$ for which

$$\text{var}\left(\lambda_{i}\right)\leq \text{max}\left[\text{var}\left(\lambda_{S}\right)\right],$$ (2)

where $i\in S_{0}$, *λ*_*i*_ corresponds to the mean prediction at *i*, S denotes the set of locations where data are observed, and $\lambda_{S}$ denotes predictions at S.

Generalizations of the linear model are often written in the form

$$Y_{i}∼f_{Y}\;\left(g^{-1}\left(\mu_{i}\right)\right),$$ (3)

where *f*_*Y*_ denotes a probability density or mass function (e.g., Bernoulli, Poisson), *g* gives a link function, and *μ*_*i*_ is a linear predictor. For many such generalizations, it is possible to specify the *μ*_*i*_ as

$$\mu =X_{aug}\beta_{aug},$$ (4)

where **X**_*aug*_ denotes an augmented design matrix, and ***β***_*aug*_ denote an augmented vector of parameters. For instance, in a spatial model, ***β***_*aug*_ might include both fixed effect parameters and spatial random effects in a reduced dimension subspace (see S1 Text for examples of how numerous types of models can be written in this form).

When models are specified as in Eq 4, we can write prediction variance generically as

$$\text{var}\left(\hat{\mu }\right)=X_{aug}\text{var}\left({\hat{\beta }}_{aug}\right)X_{aug}^{\prime },$$ (5)

where it is understood that the exact form of **X**_*aug*_ and $\text{var}({\hat{\beta }}_{aug})$ depends on the model chosen (i.e., GLM, GAM, or STRM; S1 Text). Alternative model structures for ***μ*** can also be accommodated; for instance, in Bayesian models var(***μ***) can be set equal to posterior predictive variance, $\text{var}(\tilde{\mu }|Y,\theta )$ (where ***θ*** represent hyperparameters).

The expression for prediction variance in Eq 5 is on the linear predictor scale. If a non-identity link function is used, an additional step is needed to convert prediction variance to the response scale (i.e., to calculate var(***λ***) as needed to define the gIVH in Eq 2). One approach for calculating variance on the response scale is simply to use the delta method [10, 11]. In particular, we can write the variance of the expected responses as

$$\begin{array}{ccc} \text{var}(\hat{\lambda }) & = & \text{var}(g\left(\hat{\mu }\right))\\ & \approx & \Delta \text{var}\left(\hat{\mu }\right)\Delta^{\prime }, \end{array}$$ (6)

where **Δ** is a matrix of partial derivatives of the function *g*(***μ***) with respect to its parameters, evaluated at the estimators, $\hat{\mu }$. Specifically, the *r*th row and *c*th column of **Δ** is given by $\Delta_{rc}=\partial g\left(\mu_{r}\right)/\partial \mu_{c}{|}_{\mu =\hat{\mu }}$. Under common univariate link functions (e.g., log, logit, probit), **Δ** has a diagonal form, while for multivariate links (e.g., multinomial logit) **Δ** will be dense.

Alternatively, one can use a simulation based method for determining variance of the predictive mean response vector. In Bayesian analysis of hierarchical models, this is easily accomplished via posterior predictive inference [12]. In a similar spirit, it is also possible to use parametric bootstrapping instead of the delta method to approximate prediction variance on the response scale for frequentist models [13–15].

We propose to use the gIVH in much the same manner as Cook [16]. In particular, we use the gIVH to determine whether spatial predictions are interpolations (predictive design points lying inside the gIVH) or extrapolations (predictive design points lying outside the gIVH). For most of the following treatment, we shall assume that data have already been collected (see Discussion for comments on the potential use of the gIVH in survey planning). For further details on how the gIVH was calculated for specific models in this paper, see S1 Text.

### Computing

We developed a package SpatPred in the R statistical programming environment [17] to simulate data and conduct all analyses. The seal dataset is included as part of this package, and is available at https://github.com/pconn/SpatPred/releases. The R package has also been archived via figshare [18].

### Simulation study

We conducted a simulation study to investigate whether the gIVH (and accompanying prediction variance) was useful in diagnosing prediction biases when analyzing animal count data. For each of 100 simulations, we generated animal abundance over a 30 × 30 grid assuming that animal density was homogeneous in each grid cell. Animal abundance was generated as a function of three hypothetical spatially autocorrelated habitat covariates (S2 Text). For each simulated landscape, we conducted virtual surveys of *n* = 45 survey units using two different designs: (1) a spatially balanced sample [19], and (2) a convenience sample where the probability of sampling was greater for cells closer to a “base of operations” located in the middle of the survey grid. The former approach preserves randomness while seeking a degree of regularity when distributing sampling locations across the landscape, while the latter may be easier to implement logistically.

We configured virtual sampling quadrats such that they encompassed 10% of the area of each selected grid cell. For ease of presentation and analysis, we assumed detection probability was 1.0 in each quadrat. Once animal counts were simulated, three different estimation models were fitted to the data: a GLM, a GAM, and an STRM (S1 Text). The fixed effects components of the GLM and STRM were configured to have both linear and quadratic covariate effects and first-order interactions, while the GAM expressed log-density as a function of smooth terms for each covariate (S2 Text). Each model was provided with two of the three covariates used to generate the data.

**Fig 2:**
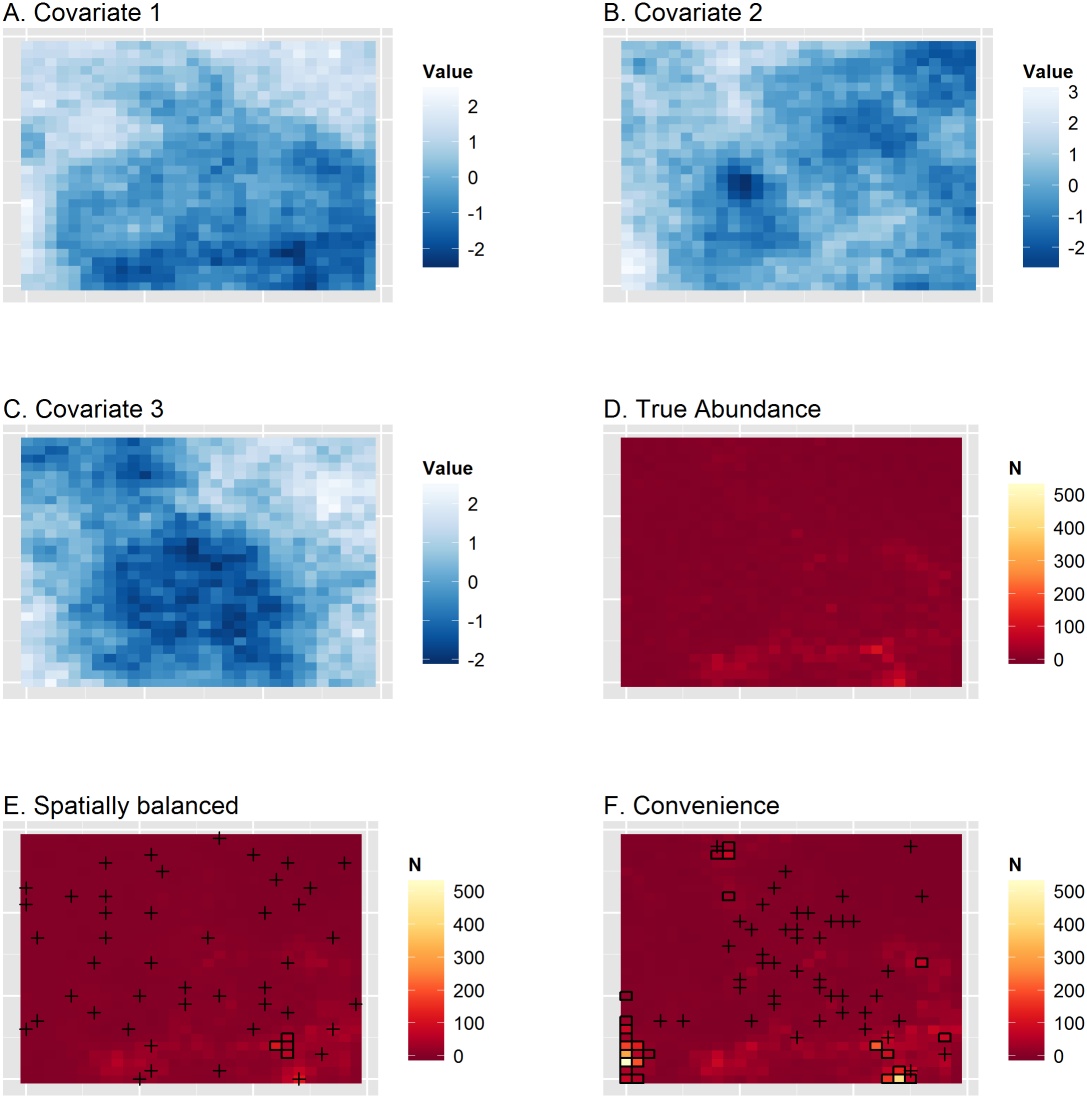
Depiction of a single simulation replicate where problematic extrapolation occurs. Panels (A-C) give simulated covariate values, panel D gives true animal abundance, (E) gives estimated abundance from a GLM run on count data from a spatially balanced survey design, and (F) gives abundance from a GLM applied to count data from a convenience survey. In (E-F), predictions outside the gIVH are represented by black boxes, and sampling locations are represented with an x. For the convenience sample, there was considerable positive bias, particularly in cells outside of the gIVH. In this case, the median posterior abundance prediction for the entire survey area is 57% greater than true abundance when inference is made to the whole study area. When inference is restricted to cells within the gIVH, median posterior abundance is 16% greater than true abundance.

For each simulated data set and model structure, we calculated the posterior predictive variance and resulting gIVH as in Eq 2. We then calculated posterior predictions of animal abundance within and outside of each gIVH in order to gauge bias as a function of this restriction. Specifically, the performance of the gIVH may help decide its utility in limiting the scope of inference once data have been collected and analyzed, and perhaps point out areas worthy of additional sampling. A fuller, technical description of the simulation study design is provided in S2 Text; a visual depiction of a single simulation replicate is displayed in Fig 2.

### Ribbon seal SDM

As part of an international effort, researchers with the U.S. National Marine Fisheries Service conducted aerial surveys over the eastern Bering Sea in 2012 and 2013. Agency scientists used infrared video to detect seals that were on ice, and collected simultaneous digital photographs to provide information on species identity. For this study, we use spatially referenced count data from photographed ribbon seals, *Phoca fasciata* on a subset of 10 flights flown over the Bering Sea from April 20–27, 2012. We limited flights to a one week period because sea ice melts rapidly in the Bering Sea in the spring, and modeling counts over a longer duration would likely require addressing how sea ice and seal abundance changes over both time and space [20]. However, limiting analysis to a one week period makes the assumption of static sea ice and seal densities tenable [21].

**Fig 3:**
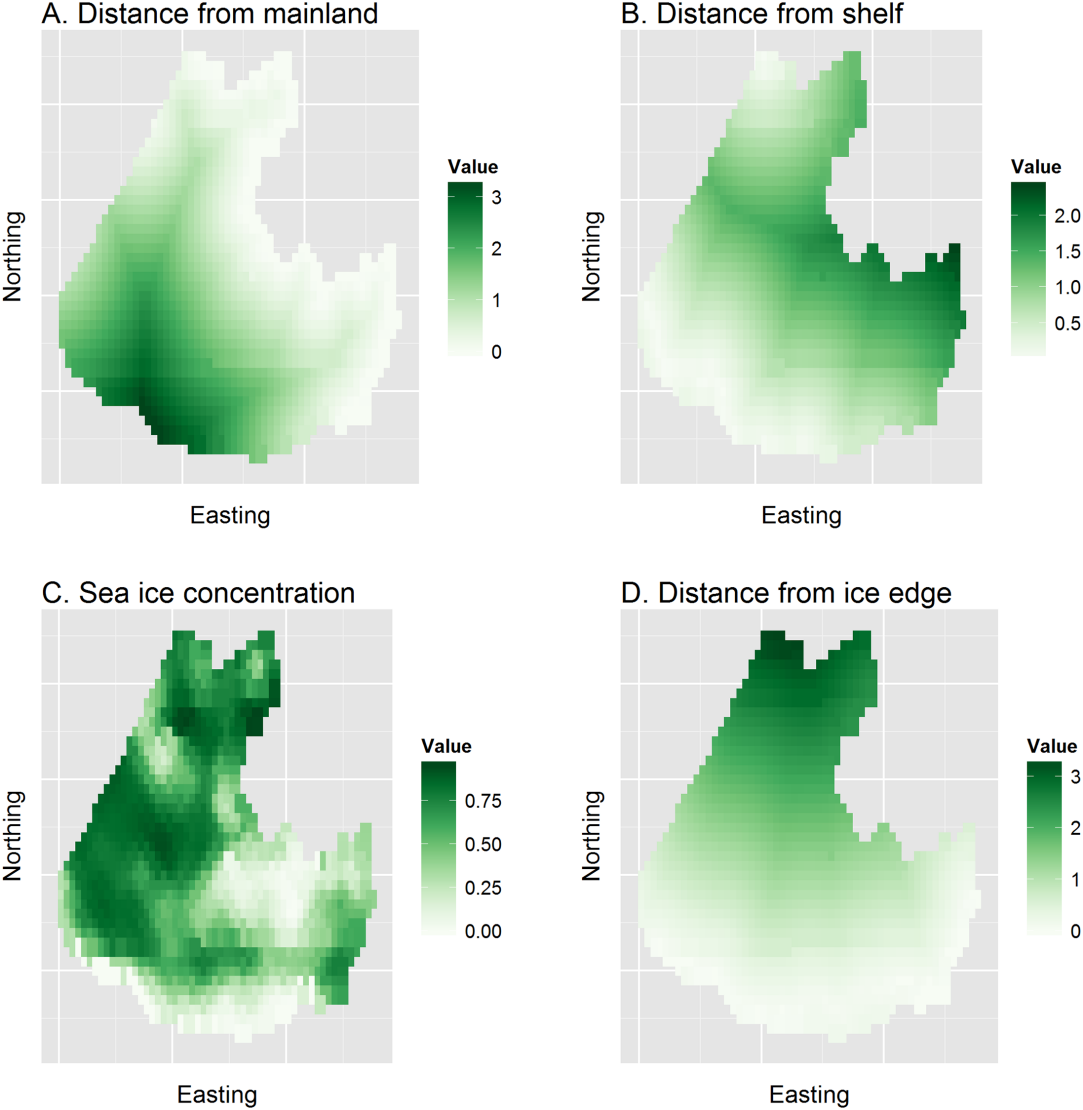
Assembled covariates used to help explain and predict ribbon seal relative abundance in the eastern Bering Sea. Covariates include distance from mainland (dist_mainland), distance from 1000m depth contour (dist_shelf), average remotely sensed sea ice concentration while surveys were being conducted (ice_conc), and distance from the southern sea ice edge (dist_edge). All covariates except ice concentration were standardized to have a mean of 1.0 prior to plotting and analysis.

Our objective with this dataset will be to model seal counts on transects through 25km by 25km grid cells as a function of habitat covariates and possible spatial autocorrelation. Estimates of apparent abundance can then be obtained by summing predictions across grid cells. Fig 3 show explanatory covariates gathered to help predict ribbon seal abundance. These data are described in fuller detail by [21], who extend the modeling framework of STRMs to account for incomplete detection and species misidentification errors. Since our focus in this paper is on illustrating spatial modeling concepts, we devote our efforts to the comparably easier problem of estimating apparent abundance (i.e., uncorrected for vagaries of the detection process).

**Fig 4:**
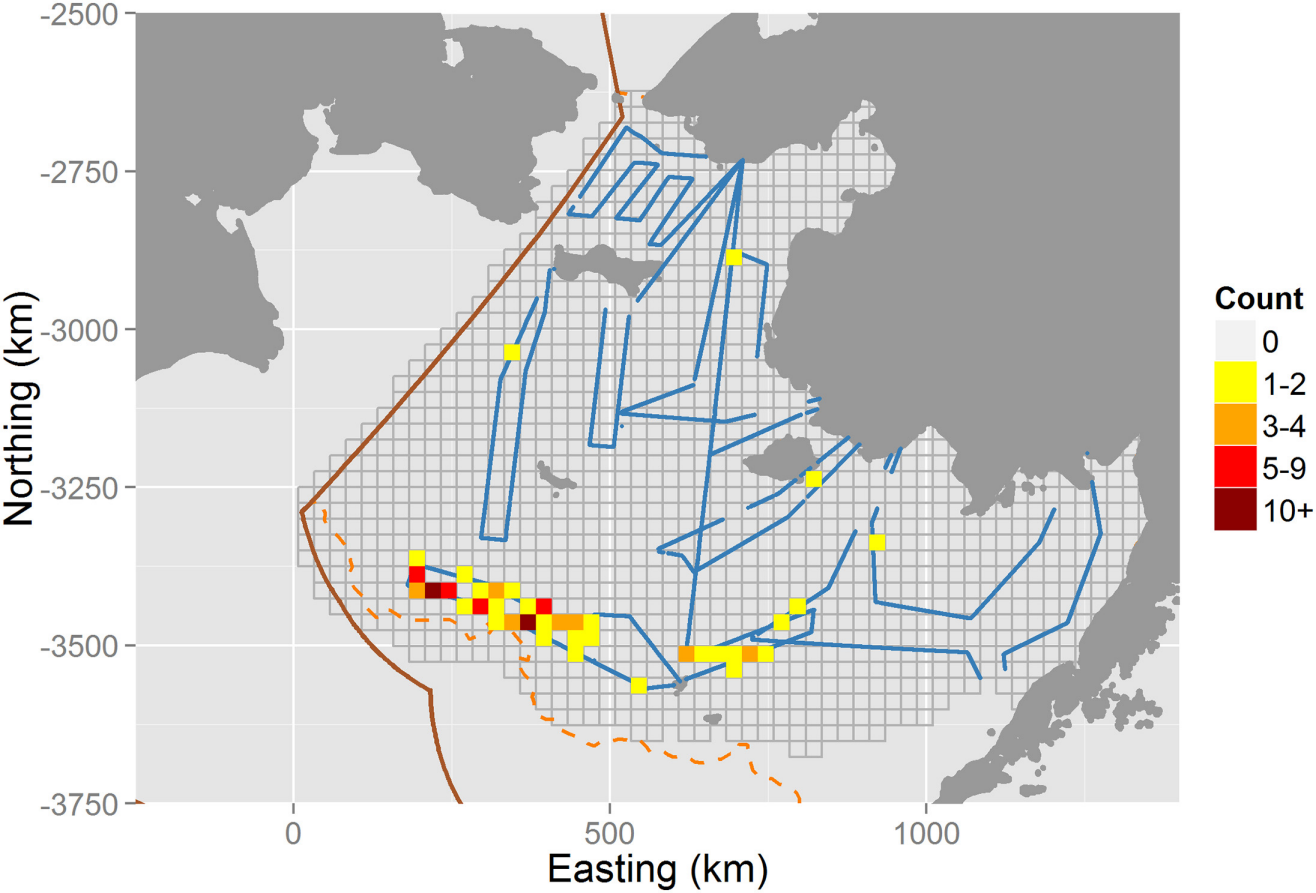
Aerial survey tracks over the Bering Sea, April 22–29, 2012. Survey tracks are shown in blue, and are overlayed on a tesselated study area consisting of 25km by 25km grid cells (gray lines). Dark gray indicates land, while the orange dashed line indicates a 1000m depth contour, and the solid brown line shows the U.S Exclusive Economic Zone (EEZ) boundary. Colored pixels indicate ribbon seal counts along aerial transects. The average effective area surveyed in each grid cell was approximately 2.6km^2^ (0.4%). Note that surveys were designed to target multiple seal species, several of which had high densities further north (results not shown).

Inspection of ribbon seal data (Fig 4) immediately reveals a potential issue with spatial prediction: abundance of ribbon seals appears to be maximized in the southern and/or southeast quadrant of the surveyed area. Predicting abundance in areas farther south and west may thus prove problematic, as the values of several explanatory covariates (Fig 3) are also maximized in these regions.

We start by fitting hierarchical GLMs and STRMs to the ribbon seal data. To accommodate incomplete coverage of grid cells and account for non-target habitat, we adapted Eqs 3 and 4 as follows. First, let *Y*_*i*_ denote the ribbon seal count (*Y*_*i*_) obtained in sampled grid cell *i*. Suppose that counts arise according to a log-Gaussian Cox process, such that

$$\begin{array}{ccc} Y_{i} & ∼ & \text{Poisson}(\lambda_{i})\;\text{and}\;\\ \log(\lambda_{i}) & = & \log\left(P_{i}\right)+\log\left(A_{i}\right)+\theta_{i}+\epsilon_{i}, \end{array}$$ (7)

where *P*_*i*_ gives the proportion of area surveyed in grid cell *i*, *A*_*i*_ gives the proportion of cell *i* that is seal habitat, *θ*_*i*_ is a linear predictor, and *ϵ*_*i*_ is normally distributed *iid* error. By formulating *θ*_*i*_ differently, we can arrive at representations characteristic of GLMs and STRMs (see S1 Text).

The fixed effects component of the GLM and STRM included linear effects of all explanatory covariates (Fig 3), as well as a quadratic effect for sea ice concentration. For the STRM, we imposed a restricted spatial regression (RSR) formulation for spatially autocorrelated random effects, where dimension reduction was accomplished by only selecting eigenvectors of the spectral decomposition associated with eigenvalues that were greater than 0.5 (see S1 Text for additional information on model structure). Adopting a Bayesian perspective, we estimated parameters for these models using MCMC (see S1 Text for algorithm details and information on prior distributions) with 60,000 iterations where the first 10,000 iterations were discarded as a burn-in. We generated posterior predictions of ribbon seal abundance across the landscape as

$$N_{i}∼\text{Poisson}\left(A_{i}\lambda_{i}\right),$$ (8)

and calculated the gIVH as in Eq 2, with delta method modifications as specified in Eq 6.

We also fitted a frequentist GAM to seal data using the mgcv R package [7]. We included smooth terms for all explanatory covariates; however, owing to relative data sparsity, we provided mgcv with the smallest basis size allowable (k = 3) for the default thin plate spline smoother. We used a quasipoisson error structure in mgcv for this analysis, which was the most similar option available to the log-Gaussian Cox formulation chosen for the GLM and STRM models. For more information on the procedure used to generate parameter estimates and abundance predictions on the response scale, see S1 Text.

**Fig 5:**
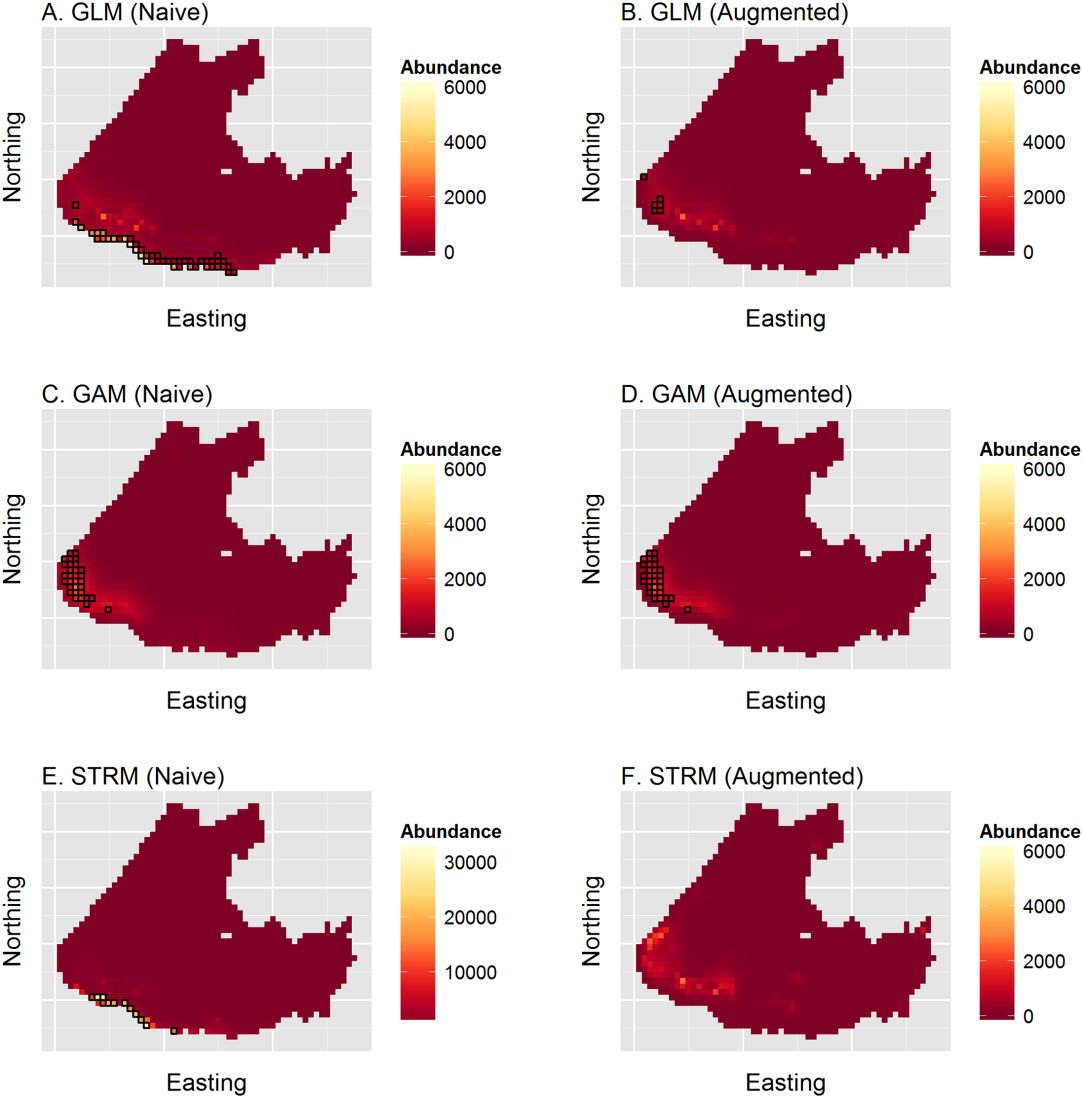
Predictions of ribbon seal apparent abundance across the eastern Bering sea from models fit to survey data. Predictions were obtained using the posterior predictive mean for GLM and STRM models, and for the GAM using the predict.gam function in the R mgcv package [7]. Each row gives result for different model types (GLM, GAM, or STRM, respectively); left column plots give results for naive runs without presumed absences, while plots in the right column give predictions for runs where presumed absence data (i.e., 0 counts in cells with <0.1% ice) were included. Cells highlighted in black indicate those where predictions were outside the generalized independent variable hull (gIVH).

Initial spatial predictions using two of the three models (GLM, STRM) produced extremely high, unbelievable predictions along the southern boundary of the study area (Fig 5). Predictions in this region were also largely out of the gIVH, indicating the potential utility for the gIVH in revealing problematic extrapolations. We considered several possible alternatives for trying to obtain more robust abundance estimates before settling on a preferred alternative. First, one could refine the study area to eliminate predictions outside of the gIVH (as in the simulation study). However, this is not ideal in that one does not get an abundance estimate for the whole study area, and it may be difficult to compare abundance from one year to the next using this approach. Second, one could try different predictive covariate models (e.g., by altering the combination or polynomial degree of covariates included in the model). Finally, one could build in a priori knowledge of habitat preferences into the model structure. We adopted the latter solution, incorporating presumed absences (i.e., zero counts where sampling was not conducted) in locations where it would have been (nearly) impossible to detect seals. Specifically, we inserted presumed absences in cells where ice concentrations were <0.1%. This solution seemed the most logical, as many of the large, anomalous predictions were over open water along the southern edge of the study area, where we would have obtained zero counts had they been surveyed. This approach effectively requires that sea ice concentration be included as a predictive covariate to help model absences in cells without ice.

## Results

### Simulation study

**Fig 6:**
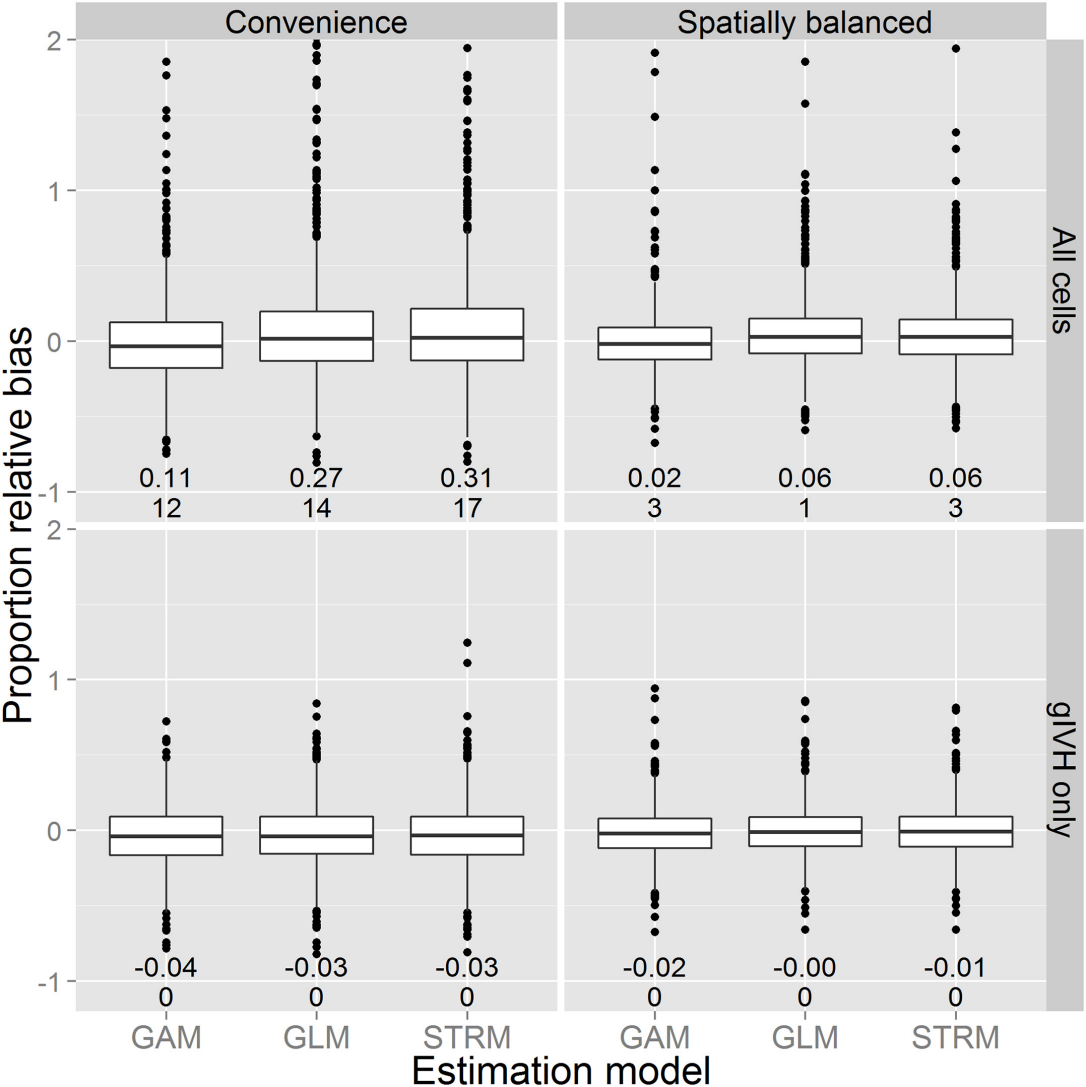
Boxplots summarizing proportional error in abundance from the simulation experiment. Each boxplot summarizes the distribution of proportional error in the posterior predictive median of abundance as a function of estimation model (x-axis), survey design (columns) and whether or not inference was restricted to the gIVH (rows). The lower and upper limits of each box correspond to first and third quartiles, while whiskers extend to the lowest and highest observed bias within 1.5 interquartile range units from the box. Outliers outside of this range are denoted with points. Horizontal lines within boxes denote median bias. The two numbers located below each boxplot indicate mean bias (upper number) and the number of additional outliers for which proportional bias was greater than 2.0 (lower number).

Posterior predictions from simulations indicated that the distribution for proportional error in total abundance was right skewed when statistical inference was made with regard to the entire survey area (Fig 6). Although median bias was close to zero, this right skew translated into positive mean bias, and was exacerbated when convenience sampling was employed. The magnitude of mean absolute bias was either the same or reduced (often substantially so) when inference was constrained to the gIVH. Positive proportional bias was the rule, and was of concerning magnitude (≈ 0.3) for GLMs and STRMs when convenience sampling was employed and inference was not restricted to the gIVH. By contrast, proportional bias was close to zero when inference was restricted to the gIVH, although there appeared to be a small negative bias (Fig 6). Interestingly, bias for frequentist GAMs was of smaller magnitude than the Bayesian GLM or STRM models for the particular model structures used here.

### Ribbon Seal SDM

Fitting our three ribbon seal SDMs to the augmented dataset with presumed absences, most predictions occurred within the gIVH (Fig 5). Posterior summaries of abundance across the entire study area were of similar magnitude, with 5%, 50%, and 95% posterior prediction quantiles as follows: GLM (48,686, 64,836, 93,927); STRM(41,039, 63,717, 194,095). The GAM produced an estimate of 92,277 (90% bootstrap CI: 63,090, 129,367). The largest differences among the three models was in the southwest corner of the study area in the area where predictions often occurred outside the gIVH. Restricting comparison of abundance to those cells that occur within the gIVH in all three models (i.e., only cells not highlighted in the right column of Fig 5), posterior prediction quantiles for the GLM were (41,750, 52,863, 69,557) and for the STRM were (39,446, 56,520, 135,427); estimated GAM abundance from mgcv was 59,104 (90% bootstrap CI:52,629, 73,076). There thus appears to be substantial between-model variation in predicted abundance when summed over the entire study area, but much better agreement (albeit with a heavier right tail for the STRM) when restricting inference to locations where predictions occur within the gIVH.

We note that these estimates are for example illustration only, as they are uncorrected for imperfect detection (e.g., incomplete detection of thermal cameras, animals that were unavailable for sampling because they were in the water, species misidentification; [21]). Our approach here was to examine extrapolation and prediction error using relatively simple models, with the understanding that such effects are also likely to occur in complex models with more realistic observation processes. Standard diagnostics (e.g., q-q plots in mgcv) also suggested some lack of fit associated with the quasipoisson error distribution; future work should investigate alternate error structures such as the Tweedie distribution [15]. Although not reported here, additional model fitting suggested sensitivity to model complexity and choice of basis, both of which are worthy of additional investigation.

## Discussion

We have demonstrated the capacity of certain classes of statistical models to produce biased predictions of animal abundance when extrapolating past the range of observed data. In simulations, commonly used models exhibited substantial mean positive bias when predictions were required for the entire study area, particularly when convenience sampling was employed. Median bias in the simulation study was close to zero, but the bias distribution was right skewed, indicating the possibility of considerably biased overestimates in a substantial proportion of simulation replicates. By contrast, restricting inference to locations within the gIVH led to small negative bias. Although this negative bias is undesirable, it may be preferable from a conservation and management standpoint. For instance, making management decisions (e.g., harvest, restoration efforts) based on estimates that have a small negative bias are much less likely to lead to catastrophic population collapse than are decisions based on overestimates.

In the ribbon seal example, naive extrapolation of fitted statistical relationships produced high positive bias along the southern boundary of the study area for the GAM and STRM models. However, the gIVH appeared useful in diagnosing places where extrapolations from the fitted statistical model were problematic. For ribbon seal relative abundance, it was useful for confirming that the naive models needed to be reformulated. Reformulated models (with presumed absence data) still yielded estimates of total abundance with considerable between-model variation in the southwest corner of the study area. However, when inference was restricted to locations within the gIVH for all three fitted models, abundance estimates were quite comparable.

When estimating species distributions, researchers often stress the need for prediction locations to be similar to the locations used for model development [22]. One way to accomplish this is through a prediction envelope, whereby a specific criterion is used to limit predictions of animal density or occurrence to the range of conditions and covariates encountered during surveys [23]. Using the gIVH for this purpose will likely be more conservative than envelope specifications based on other criterion (e.g., in contrast to minimum and maximum observed covariate values as in [23]), but is more in line with linear modeling theory. A comparison of envelope specification methods is beyond the scope of this paper, but we suspect there are cases where seemingly intuitive envelope strategies result in problematic extrapolations, particularly when the form of prediction models is of high dimension or includes multiple interaction terms.

In SDMs and model-based abundance estimation, the goal for analysts is often to build predictive maps of species abundance or occurrence using a limited number of sample locations. In such applications, the ultimate aim of analysts should be to build models that have low bias and high precision. However, traditional approaches to quantifying bias (e.g., goodness-of-fit statistics) only work with observed data points. When inference is extended to unsampled locations, the gIVH appears to be a useful diagnostic for whether bias for predictions in unsampled locations can be expected. In some cases, biological knowledge and intuition may be sufficient to diagnose anomalous predictions. However, such determinations are likely to be quite subjective, and may prove insufficient when there are a large number of regression coefficients and interaction terms. For instance, even relatively simple regression models may exhibit non-intuitive patterns (e.g., Fig 1). Further, relying on expert opinion alone in successive rounds of model formulation and fitting may lead to investigators choosing models based on how much they like the results, which is clearly not ideal scientific practice.

Our intent is to raise awareness of potential problems with extrapolation bias in statistical models, and to provide an additional tool (the gIVH) to help diagnose its presence. Other methods for selecting models to enhance predictive performance, such as cross validation [24], are also useful for this purpose, but may not entirely eliminate the problem (particularly for sparse datasets). One approach that might be useful in practice is to combine the cross validation and gIVH paradigms—for instance, using cross validation to narrow down the field to a suite of models with good predictive performance at test locations, and then calculating the gIVH to examine the potential for anomalously high predictions in unsampled locations.

The analyses in this paper focused on abundance estimation, which is necessarily non-negative. As such, counts are usually analyzed with a log link function, and there is a much greater potential for positive bias than negative. Since prediction variance tends to increase as a function of the magnitude of the prediction, the gIVH will only tend to be able to diagnose predictions that are anomalously large. However, one could also apply the gIVH when predicting species occurrence from presence/absence data. In this case, common link functions (e.g., probit or logit) are symmetric, and potential for positive and negative bias in predictive maps seem equally likely. Future research should be directed to examine conditions under which the gIVH is a useful diagnostic in such applications.

One area that gIVH ideas may also prove useful is in formulating survey designs. The topic of optimal (or near-optimal) spatial design has received considerable attention in the statistical literature, often in the context of designing environmental monitoring programs [25]. Optimal designs can be sensitive to the structure of the estimation model that is used, so that tailoring a survey design to a particular model can be somewhat dangerous if there is uncertainty about the ultimate “best” structure for the model used to relate animal abundance and occurrence to available covariates. Nevertheless, one could still think about augmenting a given sampling design with a number of locations which are known or thought to have high prediction variance as a function of available covariates (e.g., the southwest corner of the study area in the ribbon seal example). This could potentially be done as an exercise before any data (or perhaps data from a pilot study) have been collected. We are excited about this prospect, and it is a subject of current research.

## Supporting Information

S1 Text Model formulation, Gibbs sampling algorithms, and gIVH calculations for certain classes and extensions of the generalized linear model. (PDF)

S2 Text Full details of simulation study examining predictive extrapolation. (PDF)
