# Supplementary material for: On Extrapolating Past the Range of Observed Data When Making Statistical Predictions in Ecology
Source: PLoS One. 2015 Oct 23;10(10):e0141416. doi: 10.1371/journal.pone.0141416 (PMC4619888; doi:10.1371/journal.pone.0141416)
Supplement: S1 Text — (PDF) [file pone.0141416.s001.pdf]

S1 Text: Model formulation, Gibbs sampling algorithms, and gIVH calculations for certain classes and extensions of the generalized linear model

In this paper, we restrict consideration to statistical models for ecological prediction to those that can be written with the form

$$Y_i \sim f_Y(g^{-1}(\mu_i)), \tag{S1.1}$$

where $\mu_i = \xi_i + \epsilon_i$, f_Y denotes a probability density or mass function (e.g. Bernoulli, Poisson), g gives a link function, and ξ_i is a linear predictor. Many models can be fit with $\epsilon_i = 0$ (such as many frequentist generalized linear and generalized additive models), but the Bayesian models that we develop subsequently involve the specification $\epsilon_i \sim \text{Normal}(0, \tau_\epsilon)$ where τ_ϵ is a precision parameter. This specification is doubly stochastic, in the sense that we assume error associated with f_Y as well as in the location parameter μ_i. This setup can be useful computationally, and can also be used to approximate singly stochastic system by setting τ_ϵ to a large value. For instance, models for count data often assume a Poisson error structure with a log link function to ensure that the Poisson intensity parameter is greater than zero. In this case, we would specify

$$Y_i \sim \text{Poisson}(\exp(\xi_i + \epsilon_i)),$$

a configuration known as a log-Gaussian Cox process. We now describe how different classes of statistical models can be developed depending on how one structures the linear predictor, ξ_i.

1 Models

1.1 Generalized linear models (GLMs)

Generalized linear models (McCullagh and Nelder 1989) are one of the simplest (and most often used) statistical models used by ecologists to make spatial predictions. In GLMs, the linear predictor is a simple linear function of gathered covariates (including possible polynomial terms and interactions). Statisticians often describe this relationship by $\boldsymbol{\xi} = \mathbf{X}\boldsymbol{\beta}$, which is written in matrix notation. In particular, $\boldsymbol{\xi}$ gives a vector of linear predictor values (one for each data point being analyzed), $\boldsymbol{\beta}$ gives a vector of regression coefficients, and $\mathbf{X}$ is a design matrix which includes all explanatory variables (and often a column vector of ones to represent an intercept).

1.2 Generalized additive models (GAMs)

Although one can allow nonlinear relationships between response variables and regressors by including polynomial terms in the design matrix of a GLM, these need to be pre-selected by the analyst and it is often unclear how many such terms one should include. Generalized additive models (GAMs; Hastie and Tibshirani 1999, Wood 2006) build upon generalized linear models, but instead allow smooth relationships between the dependent and independent variables using flexible functions such as splines. Such models have been employed in a number of spatial prediction scenarios, including transect sampling models for animal abundance (Hedley and Buckland 2004) and SDMs (Guisan et al. 2002). For instance, animal density or presence can be modeled as a smooth, unknown function of a habitat covariate.

There are a number of ways smooth relationships can be modeled, depending upon the type of basis function employed (e.g. cubic smoothing or thin plate splines). Regardless of basis choice, it is often possible to write GAMs by substituting the following linear

predictor into Eq. S1.1:

$$\boldsymbol{\xi} = \mathbf{X}\boldsymbol{\beta} + \mathbf{K}\boldsymbol{\alpha} \tag{S1.2}$$

Here, the component $\mathbf{X}\boldsymbol{\beta}$ correspond to fixed effects in the usual GLM sense (which may or may not be modeled), $\mathbf{K}$ represents a smooth basis matrix, and $\boldsymbol{\alpha}$ is a vector of additional parameters. Our main point in writing the GAM as in Eq. S1.2 is to emphasize the structural similarity between GLMs and certain classes of GAMs. In particular, we can rewrite Eq. S1.2 as

$$\boldsymbol{\xi} = \mathbf{X}_{aug}\boldsymbol{\beta}_{aug}, \tag{S1.3}$$

where $\mathbf{X}_{aug} = [\mathbf{X} \;\; \mathbf{K}]$ (i.e., concatenating $\mathbf{X}$ and $\mathbf{K}$ horizontally), and $\boldsymbol{\beta}_{aug} = [\boldsymbol{\beta} \;\; \boldsymbol{\alpha}]'$ (the subscript *aug* denotes augmentation).

1.3 Introducing spatial and/or temporal autocorrelation: spatio-temporal regression models (STRMs)

The previous two modeling frameworks (GLMs and GAMs) do not acknowledge spatial autocorrelation above and beyond that induced by modeled covariates. However, it is common for residuals from GLM and GAM model fits to include spatial autocorrelation, which violates their common assumption of independently distributed error (Legendre 1993, Lichstein et al. 2002). GLMs and GAMs that display residual autocorrelation should be interpreted with caution, as they will tend to have overstated precision and may even be biased. In such situations, analysts often employ spatial regression models which explicitly account for spatial autocorrelation above and beyond that explained by modeled covariates.

There are a variety of ways spatial autocorrelation can be included in regression models, depending on (i) the spatial support (i.e., continuous vs. discrete), and (ii) the

particular mechanism used to impart correlation. Here, we shall focus on areal models for discrete spatial support, as our impression is that these are more commonly employed in ecological studies. Such models require that data are aggregated at the level of some sample unit (plots, grid cells, etc.). Spatio-temporal regression models (STRMs) for areal data are often specified in a similar fashion to GLMs and GAMs:

$$\boldsymbol{\xi} = \mathbf{X}\boldsymbol{\beta} + \boldsymbol{\eta}, \tag{S1.4}$$

where $\boldsymbol{\eta}$ represent spatially autocorrelated effects. In this treatment we shall limit consideration one approach for inducing spatial autocorrelation in $\boldsymbol{\eta}$: restricted spatial regression (RSR; Reich et al. 2006, Hodges and Reich 2010, Hughes and Haran 2013).

The RSR approach to spatial regression uses a reduced-rank version of the popular intrinsic conditionally autoregressive (ICAR; Besag and Kooperberg 1995, Rue and Held 2005) model for spatial random effects, reparameterized so that basis vectors are orthogonal to the main effects of interest. This approach has generated substantial recent interest, as fixed effects retain primacy in explaining variation in the ecological process of interest and problems with spatial confounding between fixed and random effects are eliminated. As such, spatial random effects are only used to account for residual autocorrelation (Reich et al. 2006, Hodges and Reich 2010) and the decision to incorporate spatial autocorrelation has little effect on the point estimates of fixed effects. In addition, reduced dimension spatial models such as RSR lighten computational burden while still accounting for course-scale spatial autocorrelation (see e.g. Latimer et al. 2009, Wikle 2010, Hughes and Haran 2013). This approach works by reformulating the spatial random effects in Eq. S1.4 as $\boldsymbol{\eta} = \mathbf{K}\boldsymbol{\alpha}$, where α is a vector of m random effects in a reduced dimension subspace. The matrix $\mathbf{K}$ can be constructed as follows (Hughes and Haran 2013):

1. Define the residual projection matrix $\mathbf{P}^{\perp} = \mathbf{I} - \mathbf{X}(\mathbf{X}'\mathbf{X})^{-1}\mathbf{X}'$
2. Calculate the Moran operator matrix $\boldsymbol{\Omega} = J\mathbf{P}^{\perp}\mathbf{W}\mathbf{P}^{\perp}/\mathbf{1}'\mathbf{W}\mathbf{1}$, where J is the number

of areal survey units

3. Define $\mathbf{K}$ as an $(n \times m)$ matrix, where the columns of $\mathbf{K}$ are composed of the eigenvectors associated with the largest m eigenvalues of $\mathbf{\Omega}$. Hughes and Haran (2013) used simulation to explore a range of such values and concluded $m = 50 - 100$ should suffice for most applications.

Here, $\mathbf{I}$ an identity matrix, $\mathbf{1}$ is a column vector of ones, and $\mathbf{W}$ represents an association matrix describing the spatial neighborhood structure of sampling units. For instance, for a first order neighborhood structure, $\mathbf{W}$ would include a 1 for all rows i and columns j where sampling unit i and j are neighbors (see Rue and Held 2005, for alternative association matrices).

2 Gibbs sampling

We now describe methods for conducting Bayesian analysis for the GLM and STRM models described above (note that we used the `mgcv` R package of Wood (2006) to conduct frequentist analysis of the GAM model, as described in the next section). We utilize Gibbs sampling, characterized by cyclically sampling groups of parameters according to their so-called full conditional distributions conditionally on data and other parameters (Gelman et al. 2004). In some cases, we are able to solve for full conditional distributions analytically and to sample from known families of probability distributions; in others, we resort to Metropolis-Hastings steps.

2.1 Gibbs sampling for the GLM model

Our Gibbs sampler for the GLM model consists of three sets of parameter updates (1) regression parameters ($\boldsymbol{\beta}$), (2) error precision (τ_ϵ), and (3) latent log-density ($\boldsymbol{\mu}$).

Sampling from $[\boldsymbol{\beta}|\cdot]$

Setting the prior for the suite of regression parameters as $[\boldsymbol{\beta}] = \mathcal{MVN}(\mathbf{0}, (\tau_\beta X'X)^{-1})$ results in the full conditional distribution

$$[\boldsymbol{\beta}|\cdot] \equiv \mathcal{N}((\mathbf{X}'\mathbf{X})^{-1}\mathbf{X}'\boldsymbol{\mu}, (\mathbf{X}'\mathbf{X})^{-1}(\tau_\epsilon + \tau_\beta)^{-1}).$$

We set $\tau_\beta = 0.01$ for applications described in this paper.

<u>Sampling from $[\boldsymbol{\tau_\epsilon}|\cdot]$</u>

Specifying a conjugate Gamma(a, b) prior for the precision parameter leads to a full conditional that is also Gamma distributed:

$$[\tau_\epsilon|\cdot] \equiv \text{Gamma}(0.5n + a, 0.5\boldsymbol{\Delta}'\boldsymbol{\Delta} + b), \tag{S1.5}$$

where $\Delta = \boldsymbol{\mu} - \mathbf{X}\boldsymbol{\beta}$. For applications in this paper we set $a = 1.0$ and $b = 0.01$. This selection admits a large range of permissable parameter values while maintaining flat probability mass near the origin.

<u>Sampling from $[\boldsymbol{\mu}|\cdot]$</u>

Although $\boldsymbol{\mu}$ could potentially be integrated out of the likelihood, its inclusion allows for easier computation of full conditionals for $\boldsymbol{\beta}$ and τ_ϵ (and for other full conditionals once the model is expanded, for example, to GAMs and STRMs). During the main MCMC phase, the $\boldsymbol{\mu}$ only need to be updated for grid cells that are exposed to sampling (see a subsequent section for information on posterior prediction for details on unsampled areas). The full conditional for μ_s in a given grid cell s is given by

$$[\mu_{s,t}|\cdot] \propto \mathcal{N}(\mu_s; \mathbf{X}_s\boldsymbol{\beta}, \tau_\epsilon^{-0.5}) \times \text{Poisson}(C_s, \exp(o_s + \mu_s)),$$

where $\mathbf{X}_s$ gives the sth row of the design matrix and o_s is an offset. For our examples, quadrats are configured to represent 10% of a grid cell, cell $o_s = \log(0.1)$. We use a Metropolis-Hastings step to sample from $[\mu_{s,t}|\cdot]$.

2.2 Gibbs sampling for the GAM model

We used a Gibbs sampler for the GAM model that looks very familiar to that for the GLM model.

Sampling from $[\boldsymbol{\tau_\epsilon}|\cdot]$

Assuming a Gamma(a, b) prior, the full conditional for $\boldsymbol{\tau_\epsilon}$ is once again given by Eq. S1.5. In this case, $\Delta = \boldsymbol{\mu} - \mathbf{X}\boldsymbol{\beta} - \mathbf{K}\boldsymbol{\alpha}$.

Sampling from $[\boldsymbol{\mu}|\cdot]$

The full conditional for μ_s in a given grid cell s is given by

$$[\mu_{s,t}|\cdot] \propto \mathcal{N}(\mu_s; \mathbf{X}\boldsymbol{\beta} + \mathbf{K}_s\boldsymbol{\alpha}, \tau_\epsilon^{-0.5}) \times \text{Poisson}(C_s, \exp(o_s + \mu_s)),$$

where $\mathbf{K}_s$ gives the sth row of $\mathbf{K}$.

Sampling from $[\boldsymbol{\alpha}|\cdot]$

We impose a Gaussian prior on smoothing kernel weights, $\boldsymbol{\alpha} \sim \mathcal{N}(0, \tau_\alpha^{-1})$. Like $\boldsymbol{\beta}$, the resulting full conditional is once again in closed form:

$$[\boldsymbol{\alpha}|\cdot] \equiv \mathcal{MVN}((\mathbf{K}'\mathbf{K})^{-1}\mathbf{K}'(\boldsymbol{\mu} - \mathbf{X}\boldsymbol{\beta}), (\mathbf{K}'\mathbf{K}^{-1}(\tau_\epsilon + \tau_\alpha)^{-1}).$$

We set $\tau_\alpha = 0.01$ in all subsequent applications.

2.3 Gibbs sampler for the STRM model

The Gibbs sampler for the RSR version of the STRM model consists of the following:

Sampling from $[\boldsymbol{\beta}|\cdot]$

Using the same prior as for the GLM results in the full conditional distribution

$$[\boldsymbol{\beta}|\cdot] \equiv \mathcal{N}((\mathbf{X}'\mathbf{X})^{-1}\mathbf{X}'(\boldsymbol{\mu} - \mathbf{K}\boldsymbol{\alpha}), (\mathbf{X}'\mathbf{X})^{-1}(\tau_\epsilon + \tau_\beta)^{-1}).$$

Sampling from $[\boldsymbol{\mu}|\cdot]$

The full conditional for μ_s in a given grid cell s is given by

$$[\mu_{s,t}|\cdot] \propto \mathcal{N}(\mu_s; \mathbf{X}_s\boldsymbol{\beta} + \mathbf{K}_s\boldsymbol{\alpha}, \tau_\epsilon^{-0.5}) \times \text{Poisson}(C_s, \exp(o_s + \mu_s)),$$

where $\mathbf{K}_s$ gives the sth row of $\mathbf{K}$.

Sampling from $[\tau_\epsilon|\cdot]$

The full conditional is the same as for the GAM model with the replacement $\Delta = \boldsymbol{\mu} - \mathbf{X}\boldsymbol{\beta} - \mathbf{K}\boldsymbol{\alpha}$.

Sampling from $[\boldsymbol{\alpha}|\cdot]$

In the RSR model, the implied prior for reduced dimension spatial effects is $\boldsymbol{\alpha} \sim \mathcal{MVN}(0, \tau_\eta^{-1}\mathbf{K}'\mathbf{Q}\mathbf{K})$. The resulting full conditional is

$$\begin{aligned} [\boldsymbol{\alpha}|\cdot] &= \mathcal{MVN}(\mathbf{M}, \boldsymbol{\Sigma}), \text{ where} \\ \boldsymbol{\Sigma}^{-1} &= \mathbf{K}'\mathbf{K}\tau_\epsilon + \tau_\eta\mathbf{K}'\mathbf{Q}\mathbf{K} \text{ and} \\ \mathbf{M} &= \boldsymbol{\Sigma}\tau_\epsilon\mathbf{K}'(\boldsymbol{\mu} - \mathbf{X}\boldsymbol{\beta}). \end{aligned}$$

See for example, Conn et al. (2014).

Sampling from $\tau_\eta|\cdot]$

Using a conjugate Gamma(a, b) prior on τ_η, we have

$$\tau_\eta \sim \text{Gamma}(0.5m + a, 0.5\boldsymbol{\alpha}'\mathbf{K}'\mathbf{Q}\mathbf{K}\boldsymbol{\alpha} + b).$$

Here, m is the number of $\boldsymbol{\alpha}$ parameters.

2.4 Generating posterior predictions

If all we wanted to do was to estimate regression coefficients, the previous samplers would suffice. However, we typically also wish to predict animal abundance across the landscape to come up with a total abundance estimate and produce a density map. Fortunately, this

is easy to do using posterior prediction, which consists of the following steps:

1. For each of n samples (where n is the desired posterior predictive sample size), choose an iteration of the MCMC sampler randomly with replacement (call this iteration t).

2. Label the tth sample of $\boldsymbol{\alpha}$, $\boldsymbol{\beta}$, $\boldsymbol{\tau_\epsilon}$, and $\boldsymbol{\mu}$ as $\boldsymbol{\alpha}^{(t)}$, $\boldsymbol{\beta}^{(t)}$, $\boldsymbol{\tau_\epsilon}^{(t)}$, and $\boldsymbol{\mu}^{(t)}$, respectively. Recall that $\boldsymbol{\mu}^{(t)}$ is only available for sampled grid cells.

3. For unsampled grid cells, generate $\boldsymbol{\mu}^{(t)} \sim \mathcal{N}(\mathbf{X}\boldsymbol{\beta}^{(t)} + \mathbf{K}\boldsymbol{\alpha}^{(t)}, \tau_\epsilon^{(t)})$

4. Sample $\mathbf{N}^{(t)} \sim \text{Poisson}(\boldsymbol{\mu}^{(t)})$, where $\mathbf{N}^{(t)}$ is a vector of posterior predictions across the landscape.

5. Compute a posterior prediction for total abundance as $N^{(t)} = \sum_s N_s^{(t)}$.

3 Frequentist analysis using `mgcv` package

In this section, we describe analysis using the `mgcv` R package (Wood 2006), and how the gIVH can be calculated conditioned on a fitted GAM model. In particular, we calculate that gIVH using prediction variance with the following steps:

1. Fit a GAM model using the `gam` function. For this presentation, we will assume this object is given the name "gam.fit." If the proportion of each sample unit (e.g., grid cell) that is surveyed is less than 1.0, an offset should be included in the formula object required by the `gam` function. For instance, a count model might specify a formula object such as `count ~offset(log(Offset)) + s(my.cov,5)`. In this case `Offset` is a vector holding the proportion of each sample unit that is surveyed.

2. Extract the augmented design matrix $\mathbf{X}_{aug}$ from Eq. S1.3 for observed data using the `predict.gam` function from `mgcv`. Wood (2006) calls this the "prediction matrix," and one can obtain it using the `type="lpmatrix"` option in `predict.gam`.

3. Calculate variance on the linear predictor scale as $V_\mu = \mathbf{X}_{aug} \hat{\text{var}}(\hat{\boldsymbol{\beta}}_{aug}) \mathbf{X}'_{aug}$, where $\hat{\text{var}}(\hat{\boldsymbol{\beta}}_{aug})$ is the variance-covariance matrix for estimated parameters (obtainable using `vcov(gam.fit)`).

4. Use the delta method (Dorfman 1938) to calculate the variance-covariance matrix on the response scale (call this V_{obs}). Set v_{max} equal to the maximum diagonal element of V_{obs}.

5. Assemble a new `data.frame` (call this `Pred`), which includes covariate values for all sample units (grid cells) one wants to make inference to.

6. Perform steps 2-3 from above using this new dataset (using `newdata=Pred` when calling predict.gam).

7. Use the delta method (Dorfman 1938) to calculate the variance-covariance on the response scale for all prediction locations (call this V_{pred}).

8. All sample units i for which $V_{pred}[i, i] \leq v_{max}$ are included in the gIVH.

Literature Cited

Besag, J., and C. Kooperberg. 1995. On conditional and intrinsic autoregressions. Biometrika **82**:733–746.

Conn, P. B., J. M. Ver Hoef, B. T. McClintock, E. E. Moreland, J. M. London, M. F. Cameron, S. P. Dahle, and P. L. Boveng. 2014. Estimating multi-species abundance using automated detection systems: ice-associated seals in the eastern Bering Sea. Methods in Ecology and Evolution **5**:1280–1293.

Dorfman, R. 1938. A note on the delta-method for finding variance formulae. The Biometric Bulletin **1**:129–137.

Gelman, A., J. B. Carlin, H. S. Stern, and D. B. Rubin. 2004. Bayesian Data Analysis, 2nd Edition. Chapman and Hall, Boca Raton.

Guisan, A., T. C. Edwards Jr., and T. Hastie. 2002. Generalized linear and generalized additive models in studies of species distributions: setting the scene. Ecological Modelling **157**:89–100.

Hastie, T. J., and R. J. Tibshirani. 1999. Generalized Additive Models. Chapman & Hall/CRC, Boca Raton, Florida.

Hedley, S., and S. Buckland. 2004. Spatial models for line transect sampling. Journal of Agricultural, Biological, and Environmental Statistics **9**:181–199.

Hodges, J., and B. Reich. 2010. Adding spatially-correlated errors can mess up the fixed effects you love. American Statistician **64**:325–334.

Hughes, J., and M. Haran. 2013. Dimension reduction and alleviation of confounding for spatial generalized mixed models. Journal of the Royal Statistical Society B **75**:139–159.

Latimer, A. M., S. Banerjee, H. Sang, E. S. Moshner, and J. A. Silander Jr. 2009. Hierarichical models facilitate spatial analysis of large data sets: a case study on invasive plant species in the northern United States. Ecology Letters **12**:144–154.

Legendre, P. 1993. Spatial autocorrelation: trouble or new paradigm? Ecology **74**:1659–1673.

Lichstein, J., T. Simons, S. Shriner, and K. E. Franzreb. 2002. Spatial autocorrelation and autoregressive models in ecology. Ecological Monographs **72**:445–463.

McCullagh, P., and J. A. Nelder. 1989. Generalized Linear Models. Chapman and Hall, New York.

Reich, B., J. Hodges, and V. Zadnik. 2006. Effects of residual smoothing on the posterior of the fixed effects in disease-mapping models. Biometrics **62**:1197–1206.

Rue, H., and L. Held. 2005. Gaussian Markov Random Fields. Chapman & Hall/CR, Boca Raton, Florida, USA.

Wikle, C. K., 2010. Low rank representations for spatial processes. Pages 89–106 *in* A. Gelfand, P. Diggle, M. Fuentes, and P. Guttorp, editors. Handbook of Spatial Statistics. Chapman & Hall.

Wood, S. N. 2006. Generalized additive models. Chapman & Hall/CRC, Boca Raton, Florida.
