# Supplementary material for: On Extrapolating Past the Range of Observed Data When Making Statistical Predictions in Ecology
Source: PLoS One. 2015 Oct 23;10(10):e0141416. doi: 10.1371/journal.pone.0141416 (PMC4619888; doi:10.1371/journal.pone.0141416)
Supplement: S2 Text — (PDF) [file pone.0141416.s002.pdf]

S2 Text: Full details of simulation study examining predictive extrapolation

Spatially explicit statistical models are increasingly used to estimate animal abundance and predict species distributions from count data. We employed a simulation study to examine the ability of the generalized independent variable hull (gIVH) to diagnose potential areas across the landscape where predictions of animal abundance using these models may be problematic, and to determine whether statistical inferences are more robust when restricted to sampling units within the gIVH. Each simulation replicate consisted of several steps, including

1. Simulate three hypothetical, statistically dependent, spatially autocorrelated environmental covariates over a 30×30 grid,
2. Simulate animal abundance across the landscape as a function of environmental covariates,
3. Simulate surveys across the landscape, including the position of count quadrats and the resulting animal counts,
4. Estimate animal abundance as a function of two of the environmental covariates according three different models: a generalized linear model (GLM), a generalized additive model (GAM), and a spatio-temporal regression model (STRM). For the latter, only a spatial dimension was modeled. The GLM and STRM models were specified hierarchically, with MCMC used for posterior simulation. For the GAM, we conducted a frequentist analysis using the `mgcv` R package (Wood 2006).
5. Calculate the gIVH using realized posterior variance (i.e. after data were collected and analyzed).

We now describe each of these tasks and results of the simulation study in further detail before describing results. All analyses were performed in the R programming environment

(R Development Core Team 2012); requisite code to recreate analyses is available in the R package `SpatPred` that accompanies this article.

1. Simulating environmental covariates

In the real world, different habitat covariates are often correlated with each other (e.g., altitude and precipitation), and are often patchily distributed across the landscape (i.e., are spatially autocorrelated). We thus desired a procedure for generating covariates that would allow some level of statistical dependence among covariates, together with spatial autocorrelation. For each simulation, we generated three spatially autocorrelated environmental covariates using a procedure motivated by linear coregionalization models in multivariate spatial statistics (e.g. Goulard and Voltz 1992) to impart desired behavior. To start, we used the R package `RandomFields` to simulate 10 realizations $\mathbf{y}_i$ ($i \in 1, 2, \ldots, 10$) of independent, mean-zero random fields over a 30×30 grid (where the lower case bold type denotes a vector). Each random field had a stationary, isotropic, exponential covariance structure, where the covariance C between two survey units (i.e. grid cells) was a function of the distance r between grid cell centroids, $C(r) = \exp(r/v)$. Note that the distance between horizontally and vertically adjacent grid cell centroids was standardized to 1.0; the scale parameter v of the exponential covariance function for each random field was drawn from a Uniform(5,100) distribution to induce heterogeneity in the spatial scale of each process.

Next, we determined the values of three spatially autocorrelated habitat covariates, $\mathbf{z}_j$ by writing them as linear functions of the $\mathbf{y}_i$:

$$\mathbf{z}_j = \sum_i \omega_{ij} \mathbf{y}_i.$$

Evidently, the covariance between each induced habitat covariate is a function of the weights ω_{ij}. We used the following strategy to set ω_{ij}:

1. Set all $\omega_{ij} = 0$

2. For each desired covariate, j, randomly sample four values $\mathbf{u}_j$ from the set $\{1, 2, \ldots, 10\}$ without replacement.

3. For each value $u \in \mathbf{u}_j$, set $\omega_{uj} \sim \mathcal{N}(0, 1)$.

This procedure led to "patchy" habitat covariates with realistic levels of covariation (Fig. S2.1). To prevent redundancy and collinearity, we rejected and resampled covariate values (using the same procedure) whenever maximum absolute correlation among covariates was greater than 0.75.

2. Simulating animal abundance

Given values of the three simulated covariates, we generated a vector of expected log-abundance in each cell ($\boldsymbol{\mu}$) as

$$\boldsymbol{\mu} = \beta_0 + \mathbf{X}\boldsymbol{\beta} + \boldsymbol{\epsilon},$$

where the regression coefficients $\boldsymbol{\beta}$ were each drawn from a $\mathcal{N}(0, \tau)$ distribution, and the intercept, β_0, was drawn from a $\mathcal{N}(2.5, 0.25)$ distribution. The design matrix $\mathbf{X}$ was constructed assuming linear and quadratic effects for each covariate, together with all one-way interactions for a total of 9 coefficients in addition to the intercept. The precision, τ, was set to 2.5 for linear fixed effects, and to 5.0 for quadratic effects and one-way interaction terms. Residual Gaussian errors ($\boldsymbol{\epsilon}$) were drawn from a $\mathcal{N}(0, 10)$ distribution. Abundance in each cell i was generated as

$$N_i \sim \text{Poisson}(\exp(\mu_i)).$$

Regression coefficients were redrawn whenever $\sum_i N_i > 100,000$ or if the 20 most populous grid cells included $> 90\%$ of total abundance to prevent unreasonably high or constricted distributions of abundance.

3. Simulating sample locations and count data

For each simulated landscape, we selected 45 grid cells (5%) for sampling. We employed two possible survey designs: i) spatially balanced sampling using a random tessellation design (Stevens Jr. and Olsen 2004), and ii) a convenience sample where the inclusion probability of each grid cell i in the sampling frame was set proportional to $\exp(-0.2 * r_i)$, where r_i is the Euclidean distance between the centroid of grid cell i and the center of the survey grid. The latter survey design was meant to approximate the case where there is a "base of operations" in the middle of the survey grid and more effort is expended close to the center due to simpler sampling logistics. We configured simulations such that sample quadrats covered 10% of each targeted grid cell, and generated animal counts for each quadrat j as

$$C_j \sim \text{Binomial}(N_j, 0.1).$$

4. Estimating animal abundance

For each set of count data, we attempted to estimate animal abundance over the landscape using three different models, corresponding to a hierarchical, Bayesian generalized linear model (GLM; linear, fixed effects of covariates on the log scale), a frequentist generalized additive model (GAM; smooth effects of covariates on the log scale) fit using `mgcv`, and a hierarchical spatial regression model (STRM; with both linear, fixed effects and spatially autocorrelated random effects). Further details on these models, together with the procedure used for posterior simulation, are presented in Text S1. For this study, we used the RSR implementation of the STRM outlined in Text S1. Prior distributions for each precision parameter, τ, were set to Gamma$(1.0, 0.01)$, which is diffuse while maintaining a flat shape near the origin. Regression parameters (β) were given vague $\mathcal{N}(0, \tau = 0.01)$ priors. For the GLM and STRM, we used the median posterior prediction of total abundance as a point estimator. For the GAM, we used the sum of predictions of total abundance on the response scale, obtained using the `predict.gam` function in `mgcv`.

5. Calculating the gIVH

As suggested in Text S1, we used Eq. S1.7 to calculate the gIVH for the GLM and STRM models, substituting in samples from the joint posterior distribution of each model for $\boldsymbol{\theta}$. For the GAM, we used the delta method (Dorfman 1938) to calculate prediction variance on the response scale, conditioning on estimated β values.

Literature Cited

Dorfman, R. 1938. A note on the delta-method for finding variance formulae. The Biometric Bulletin **1**:129–137.

Goulard, M., and M. Voltz. 1992. Linear coregionalization model: Tools for estimation and choice of cross-variogram matrix. Mathematical Geology **24**:269–286.

R Development Core Team, 2012. R: A Language and Environment for Statistical Computing. R Foundation for Statistical Computing, Vienna, Austria. URL http://www.R-project.org.

Stevens Jr., D., and A. Olsen. 2004. Spatially balanced sampling of natural resources. Journal of the American Statistical Association **99**:262–278.

Wood, S. N. 2006. Generalized additive models. Chapman & Hall/CRC, Boca Raton, Florida.

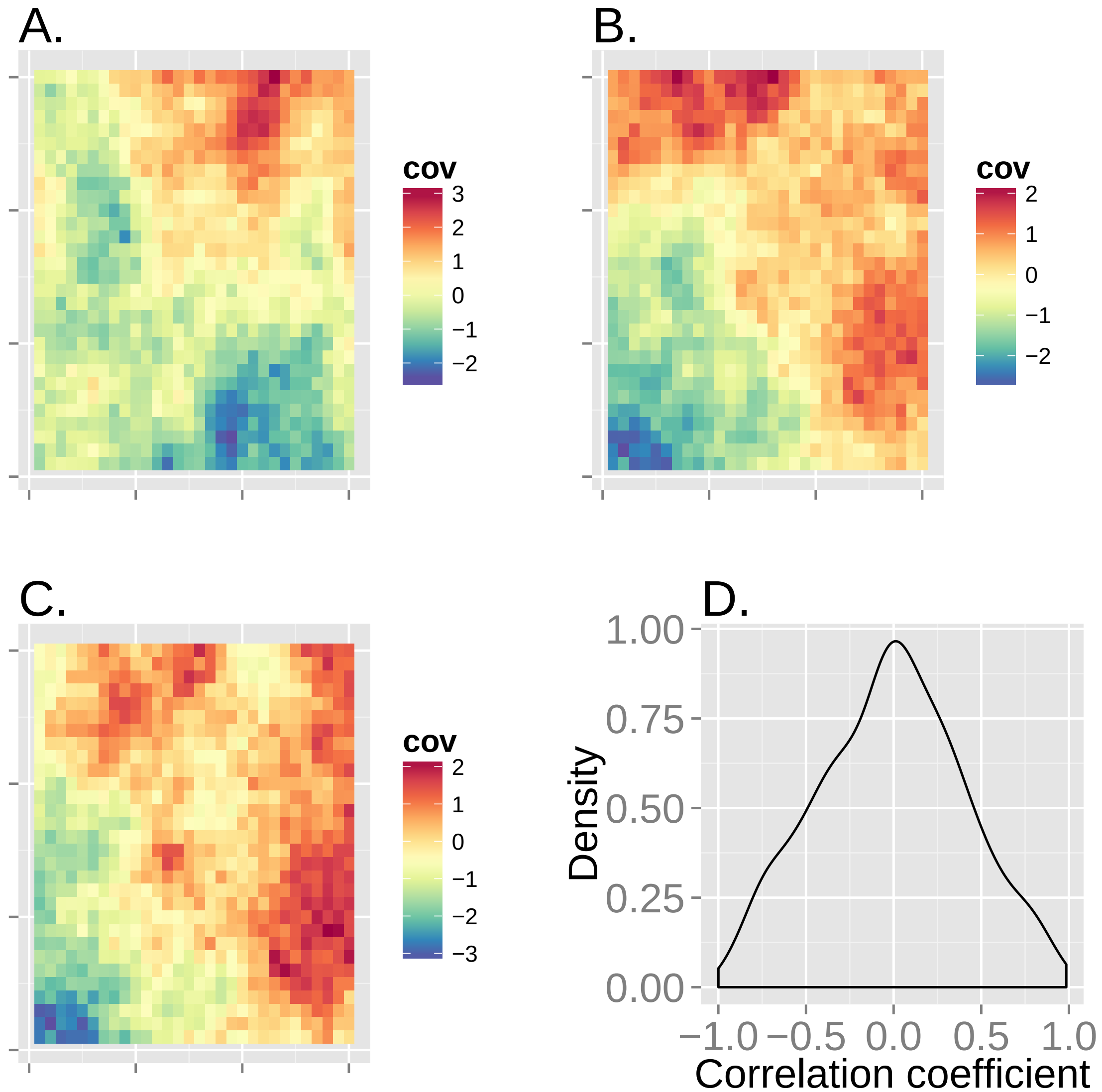

Figure S2.1. For each simulation, three spatially autocorrelated environmental covariates were generated via a linear coregionalization model. Panels A-C show a single realization from this procedure, while panel D shows the distribution of sample correlations between two randomly selected covariates over 1000 simulations.
